# Regulation of the Later Stages of Nodulation Stimulated by IPD3/CYCLOPS Transcription Factor and Cytokinin in Pea *Pisum sativum* L.

**DOI:** 10.3390/plants11010056

**Published:** 2021-12-25

**Authors:** Elizaveta S. Rudaya, Polina Yu. Kozyulina, Olga A. Pavlova, Alexandra V. Dolgikh, Alexandra N. Ivanova, Elena A. Dolgikh

**Affiliations:** 1 All-Russia Research Institute for Agricultural Microbiology, Podbelsky chausse 3, Pushkin, 196608 St. Petersburg, Russia; rudaya.s.e@gmail.com (E.S.R.); polykoz@gmail.com (P.Y.K.); dobbi85@list.ru (O.A.P.); sqshadol@gmail.com (A.V.D.); 2 Komarov Botanical Institute RAS, Prof. Popov St., 2, 197376 St. Petersburg, Russia; alyx@bk.ru; 3 Faculty of Biology, St. Petersburg State University, Universitetskaya Emb. 7-9, 199034 St. Petersburg, Russia

**Keywords:** *Pisum sativum*, transcriptomic data, IPD3/CYCLOPS transcription factor, cytokinin, gene expression

## Abstract

The IPD3/CYCLOPS transcription factor was shown to be involved in the regulation of nodule primordia development and subsequent stages of nodule differentiation. In contrast to early stages, the stages related to nodule differentiation remain less studied. Recently, we have shown that the accumulation of cytokinin at later stages may significantly impact nodule development. This conclusion was based on a comparative analysis of cytokinin localization between pea wild type and *ipd3/cyclops* mutants. However, the role of cytokinin at these later stages of nodulation is still far from understood. To determine a set of genes involved in the regulation of later stages of nodule development connected with infection progress, intracellular accommodation, as well as plant tissue and bacteroid differentiation, the RNA-seq analysis of pea mutant SGEFix^-^-2 (*sym33*) nodules impaired in these processes compared to wild type SGE nodules was performed. To verify cytokinin’s influence on late nodule development stages, the comparative RNA-seq analysis of SGEFix^-^-2 (*sym33*) mutant plants treated with cytokinin was also conducted. Findings suggest a significant role of cytokinin in the regulation of later stages of nodule development.

## 1. Introduction

Signal exchange between legume plants and rhizobial bacteria from the Rhizobiaceae family results in the development of specialized organs on roots, the nitrogen-fixing nodules. Nod factors are perceived by the complexes of transmembrane LysM-receptor-like kinases in the root epidermal cells, which trigger symbiosis development. In legumes with the determinate type of nodules such as *Lotus japonicus*, the complex of LjNFR5/LjNFR1 receptors perceives Nod factors [1,2,3], while in legumes with indeterminate nodule type, such as *Medicago truncatula* and *Pisum sativum* L., several receptor complexes can be involved in the control of early plant responses to Nod factors and infection development [4,5,6,7,8,9]. Receptor complex MtNFP/MtLYK3, MtLYK4 is involved in the control of infection process in *M. truncatula* [5,6], whereas in pea *P. sativum* L. the complex consists of PsSym10/PsSym37 and, probably, PsSym2 (PsLykX) [8,9,10]. PsSym2 (PsLykX) is required for strain-specific inoculation of pea cultivars from Afghanistan and Iran (*Sym2*^A^) and related to specific blocking of the infection process [10,11]. Early responses can be controlled by the PsSym10/PsK1 receptor complex in pea [1,9,12], while it remains unknown whether the MtNFP can function in complex with an additional receptor kinase in *M. truncatula* at the early stages of symbiosis. Subsequently, the signal is transmitted into the cell by receptor kinase with leucine-rich (LRR) repeats in the extracellular domain MtDMI2/PsSym19/LjSYMRK [13,14]. Signal transduction involves mevalonates synthesized by the enzyme 3-hydroxy-3-glutaryl coenzyme A reductase 1 (MtHMGR1) activated by MtDMI2/PsSym19/LjSYMRK. Cation channels MtDMI1/PsSym8/LjPOLLUX and LjCASTOR [15,16], the components of nuclear pore NUP85, NUP133, and NENA [17,18,19], and calcium channels MtCNGC15a,b,c/PsCNGC15a,b,c are recruited at the next stages and induce calcium spiking in the nucleus and perinuclear space [20]. In addition, the ATP-dependent calcium ATPase MtMCA8/PsMCA8/LjMCA8 is also involved in calcium exchange, promoting the removal of calcium from the nucleus [21]. Changes in nuclear calcium concentration can be perceived by calcium, calmodulin-dependent protein kinase CCaMK (MtDMI3/PsSYM9/LjCCaMK), to activate subsequent stages connected with infection and organogenesis in symbiosis [22,23,24]. Therefore, the signal cascade triggered by Nod factors leads to the realization of two genetic programs in nodule development: infection process and organogenesis [25].

Slightly different regulators may be involved in the regulation of the infection process and organogenesis and many of them remain unknown. Under the influence of СCaMK, phosphorylation of its target, the coiled-coil MtIPD3/PsSym33/LjCYCLOPS transcription factor, occurs followed by its activation [26,27,28,29]. In turn, the MtIPD3/PsSym33/LjCYCLOPS transcription factor activates the *NIN* gene by binding with a specific sequence in the promoter of this gene, the CYC-box [29,30]. NIN transcription factor is essential for rhizobial entry via infection threads in the epidermis [31,32,33,34,35]. In addition, the MtIPD3/PsSym33/LjCYCLOPS can stimulate the transcription factors from the GRAS family (MtNSP1-MtNSP2/PsNSP1-PsNSP2/LjNSP1-LjNSP2) and MYB family (IPN2), which support the signal transduction in the epidermis [36,37,38,39,40]. They can form complex and activate the *NIN* gene expression independently, through binding to another IPN2-RE responsive element in the promoter of this gene [40]. ERN1 (ERF Required for nodulation 1) transcription factor is also activated in response to calcium spiking in root hairs [41,42,43]. Both the NSP1-NSP2 complex and ERN1 transcription factors are essential for triggering the full-level expression of the *Enod11* gene encoding proline-rich cell wall protein necessary for infection development [44]. In addition, recent studies suggest that regulators of plant response to gibberellins, the DELLA proteins, interact with NSP2-NSP1 to induce the expression of ERN1 [45,46,47]. DELLA proteins can also function as a bridge linking IPD3/CYCLOPS and NSP2-NSP1 [46]. Finally, the NIN transcription factor may activate its downstream targets related to infection development: the components of heterotrimeric CCAAT-box-binding factor complex, the nuclear factor Y proteins (NF-Ys) [48,49,50]. This activation leads to the development of the infection program in epidermis cells, where the NIN, NF-YA1, and ERN1 transcription factors bind with a whole complex of additional regulators based on Chip-seq analysis [30].

In legumes with an indeterminate type of nodules, the activation of the organogenesis is related to pericycle, endodermis, and root inner cortex cells remote from the epidermis. This activation involves mobile regulators, which are activated under the influence of the NIN transcription factor in the epidermis and move into the cells of the cortex [30]. Presumably, they can be transcription factors or microRNAs, but these specific regulators have not been identified yet. However, it was shown that these unknown factors affect the accumulation of cytokinin in pericycle cells, and the regulator of the cytokinin response, the B-type RR transcription factor, which additionally stimulates the expression of the *NIN* gene in pericycle cells upon binding to the remote CE-box in the promoter of this gene [32]. In turn, the NIN transcription factor binds to the promoters of the genes encoding NF-YA1 and NF-YB1 subunits of the heterotrimeric complex of CCAAT- box-binding factors, which, through the MtLBD16/LjASL18 regulator and STY (Short internodes/Stylish) transcription factors, activate the expression of genes encoding enzymes involved in auxin biosynthesis such as YUCCA [33,51]. Auxin stimulates the subsequent proliferation of pericycle and endodermal cells followed by coordinated mitotic activation of cortical cells and nodule primordium formation. Therefore, the primary changes in cytokinin level and activation of *the NIN* gene in pericycle and endodermis cells may precede auxin accumulation in these cells and later in inner root cortical cells, which leads to primordium development. It was shown that the transcription factor MtKNOX3/PsKNOX3 stimulates cytokinin biosynthesis in developing nodules through IPT3, and LOG1, LOG2 enzymes [52]. Moreover, NIN up-regulates the expression of the *CRE1* gene encoding cytokinin receptor [53], which is required for the perception of cytokinin at the initial stages of nodulation but may also be involved in the control of later stages of nodule development [54].

In contrast to the early stages, the subsequent stages related to nodule differentiation remain less studied. Recently, we have shown that the accumulation of cytokinin at later stages may significantly impact nodule development. This conclusion was based on a comparative analysis of cytokinin localization between wild-type pea and *ipd3/cyclops* mutants [55]. However, the role of cytokinin at these later stages of nodulation is still far from understood. Here, we present data from the comparative transcriptomic analysis of the wild type and *ipd3/cyclops* mutant nodules SGEFix^-^-2 (*sym33*). SGEFix^-^-2 (*sym33*) mutant, which carries defective gene encoding IPD3/CYCLOPS transcription factor, has “locked” infection threads inside the nodule with very occasional bacterial release. This results in non-infected nodules and disturbance in subsequent plant tissue and bacteroid differentiation [56,57,58,59]. We have also estimated the effect of cytokinin treatment on this mutant using transcriptomic analysis.

## 2. Results

### 2.1. Effect of Exogenously Applied Cytokinin on the Morphology and Structural Characteristics of SGEFix^-^-2 (sym33) Mutant Nodules

At the first stage, the effect of treatment with 10 µM synthetic cytokinin (6-benzyl amino purine) BAP on *sym33* pea mutant plants was estimated. It was previously shown that concentrations of cytokinin BAP in the range of 1–10 µM may stimulate the nodulation in *M. sativa* and pea plants, while inhibition of nodule formation was shown in the range of 20–25 µM due to the stimulation of ethylene in response to treatment [60,61].

**Figure 1:**
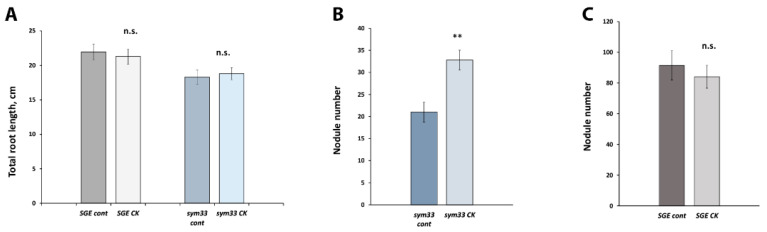
Analysis of root length (**A**) and the number of nodules in SGEFix^-^-2 (*sym33*) mutant (**B**) and wild-type cv. SGE (**C**) plants 2 weeks after inoculation with *Rhizobium leguminosarum* bv. *viciae* CIAM1026 and treatment with 10 μM 6-BAP (cytokinin, CK). The graphs show the results of three independent experiments (4–5 plants per one variant were used). The error bars represent the mean ± SEM of three repeats. The asterisks indicate statistically significant differences based on Student’s *t*-test (** *p* < 0.01).

Pea plants were harvested 14 days after inoculation (14 dai) and morphological parameters were measured. Primary and lateral roots length, as well as a number of nodules, were recorded (Figure 1A–C). Analysis revealed the increased number of nodules per plant in the SGEFix^-^-2 (*sym33*) mutant treated with 10 µM BAP (Figure 1B). At the same time, no effect on nodule number was shown for wild-type plants of cv. SGE after BAP treatment (Figure 1C). Although the cytokinin may inhibit the root development, no significant negative effect on primary root and lateral roots length was detected in wild type and mutant after 10 µM BAP application (Figure 1A). Therefore, this demonstrates that cytokinin treatment may have a stimulating effect on nodule formation in *sym33* mutant. We hypothesized that cytokinin may up-regulate the expression of genes related to further stages of nodule development in *sym33* mutant.

### 2.2. Histological Analysis of Nodules in Wild Type and sym33 Mutant Plants Treated with Cytokinin

**Figure 2:**
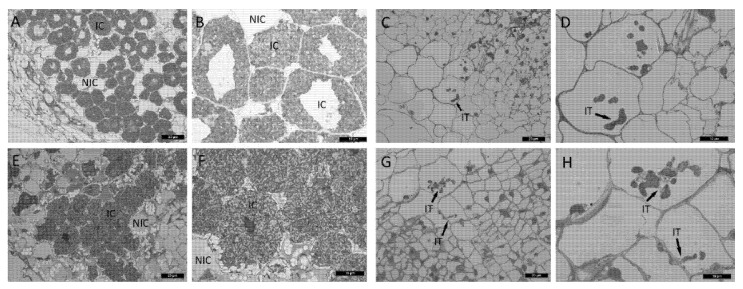
Light microscopy images of 2-week-old nontreated nodules of wild-type (**A**,**B**) and *sym33* mutant (**C**,**D**) and treated with 10 μM 6-BAP (cytokinin) wild-type (**E**,**F**) and *sym33* mutant (**G**,**H**). IC—infected cells, NIC—noninfected cells, IT—infection threads. (**A**,**C**,**E**,**G)** images have 40× magnification. (**B**,**D**,**F**,**H**) images have 100× magnification. Arrowheads indicate infection threads. Scale bars are 20 μm (**A**) and 10 μm (**B**).

**Figure 3:**
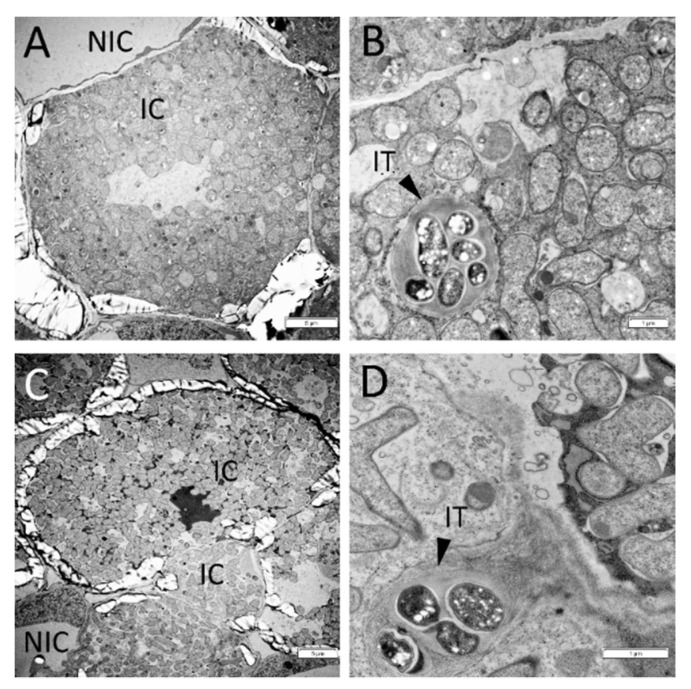
Transmission electron microscopy images of 2-week-old wild-type nodules nontreated (**A**,**B**) or treated (**C**,**D**) with 10 μM 6-BAP (cytokinin) plants. IC—infected cells, NIC—noninfected cells, IT—infection thread. Arrowheads indicate infection threads. Scale bars are 5 μm (**A**,**C**) and 1 μm (**B**,**D**).

**Figure 4:**
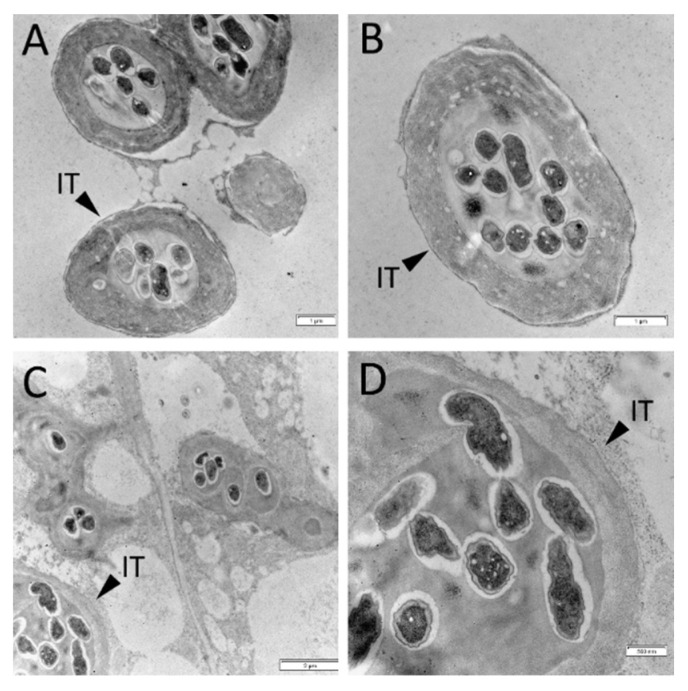
Transmission electron microscopy images of 2-week-old nodules of *sym33* mutant nontreated (**A**,**B**) or treated (**C**,**D**) with 10 μM 6-BAP (cytokinin). IT—infection thread. Scale bars are 2 μm (**C**), 1 μm (**A**,**B**), and 500 nm (**D**).

To verify the influence of cytokinin on morphological features of nodule development in wild type and mutant, light microscopy and transmission electron microscopy (TEM) were used. No significant effect of cytokinin on wild-type nodules was found (Figure 2E,F). The development of infection threads and bacteroids did not change noticeably in response to cytokinin treatment (Figure 2E,F and Figure 3). SGEFix^-^-2 (*sym33*) mutant has “locked” infection threads inside the nodule and blocking in bacterial release[56,57,58,59], which was also shown in our experiments (Figure 2C,D). Although we found that the cytokinin stimulated the additional formation of nodules in *sym33* mutant, the development of infection threads and bacterial accommodation seems to be similarly impaired in nodules of mutant plants nontreated or treated with cytokinin (Figure 2C,D,G,H). At the same time, TEM analysis showed that the development of bacteria in infection threads in the nodules of *sym33* mutant treated with cytokinin may be blocked and degradation of bacteria was sometimes visible (Figure 4).

### 2.3. Comparative Analysis of Expression Patterns between Cv. SGE Wild Type and SGEFix^-^-2 (Sym33) Mutant Nodules Using a Transcriptomic Approach

**Figure 5:**
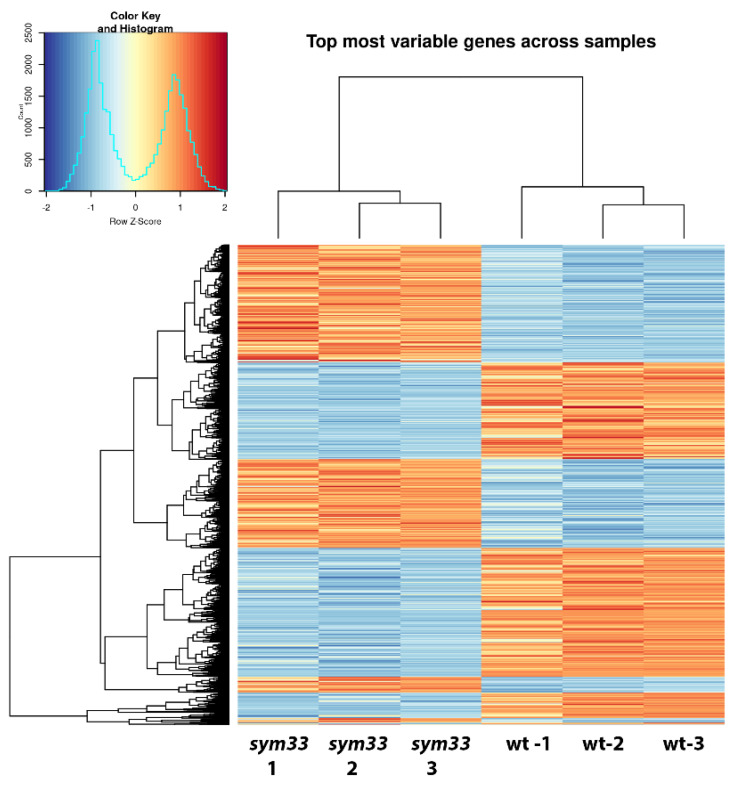
Differential gene expression in pea nodules of cv. SGE wild type and SGEFix^-^-2 (*sym33*) mutant. Heatmap represents top differentially expressed transcripts in wild-type nodules to *sym33* mutant nodules as normalized counts after variance stabilizing transformation of raw read counts per transcript. The data of three independent biological repeats were combined for each variant—*sym33* and wild type (wt) nodules.

**Figure 6:**
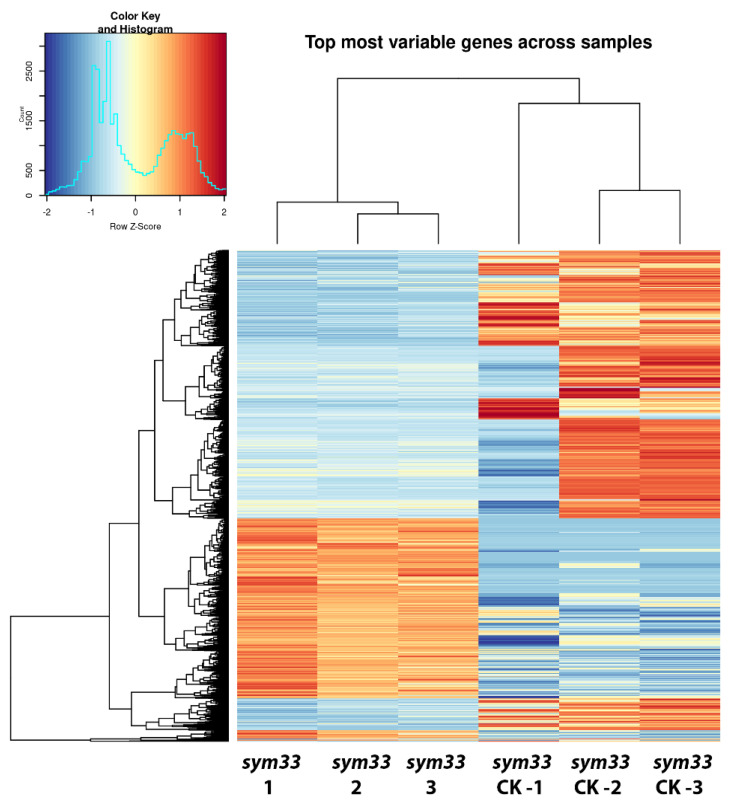
Differential gene expression in pea nodules of SGEFix^-^-2 (*sym33*) mutant plants untreated or treated with 10 µM BAP (cytokinin). Heatmap represents top differentially expressed transcripts in nodules of *sym33* mutant plants non-treated and treated with cytokinin (CK) as normalized counts after variance stabilizing transformation of raw read counts per transcript. The data of three independent biological repeats were combined for each variant—*sym33* nodules without treatment and *sym33* after cytokinin (CK) treatment.

Given that we found an increase in the number of SGEFix^-^2 (*sym33*) mutant nodules after treatment with cytokinin, as well as morphological changes in them, we decided to analyze the gene expression changes associated with these effects. We have compared the patterns of differential expressed genes between cv. SGE wild-type nodules and SGEFix^-^2 (*sym33*) mutant nodules (Figure 5) as well as between nodules of SGEFix^-^2 (*sym33*) mutant plants without treatment and treated with cytokinin (Figure 5). Using a cutoff threshold equal to log2 fold change value > 2 and *p* adjusted value < 0.05, we discovered that about 1365 genes were up-regulated in wild type SGE nodules compared with *sym33* mutant nodules, while about 2906 transcripts were up-regulated in *sym33* mutant in response to cytokinin treatment compared to unstimulated mutant (Figure S1). Moreover, 538 genes were similarly up-regulated in both sets (Figure 6). Since 538 genes represent about 40% of the total number of up-regulated genes in wild-type nodules compared with mutant (1365 up-regulated), we suggest that cytokinin plays an important role in the regulation of late stages of nodule development. These results are in line with our previous findings demonstrating significant differences in the content of cytokinin between wild-type and *sym33* mutant nodules [55].

### 2.4. Functional Activity of Differentially Expressed Genes between cv. SGE Wild Type and SGEFix^-^-2 (sym33) Mutant Based on Gene Ontology Analysis

**Figure 7:**
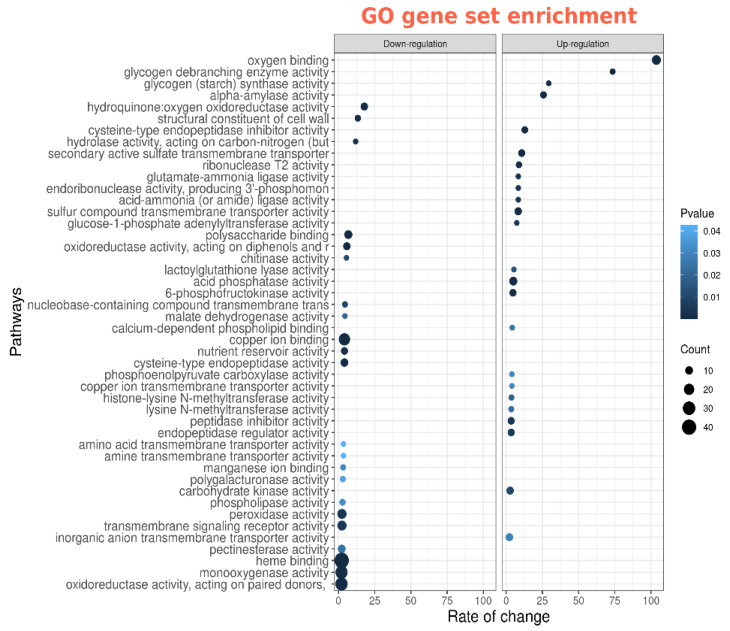
Gene ontology pathways that are overrepresented in differentially expressed genes in wild-type nodules compared to *sym33* mutant nodules (up-and down-regulated separately).

**Figure 8:**
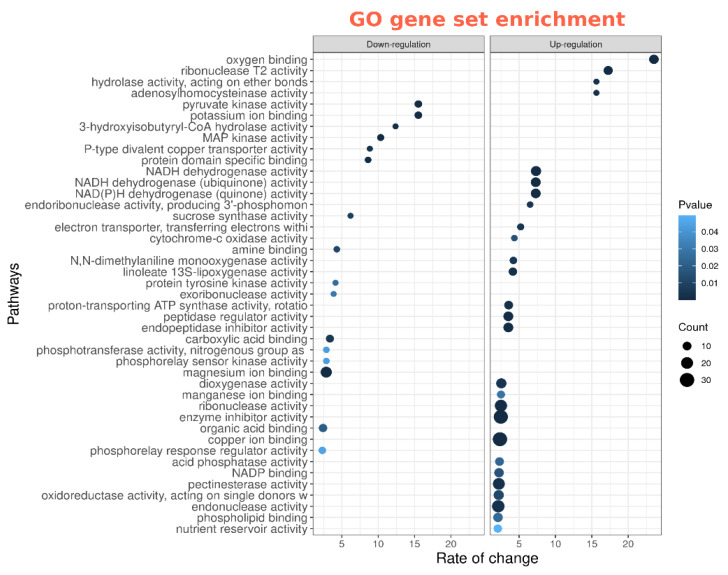
Gene ontology pathways that are overrepresented in differentially expressed genes in nodules of untreated *sym33* mutant plants compared to cytokinin treated (up-and down-regulated separately).

We used gene ontology (GO) set enrichment analysis to find out which biological processes were impacted in mutant nodules in comparison with wild-type nodules and which functions were affected in Fix^-^ nodules by cytokinin treatment (Figure 7 and Figure 8). Analysis of gene ontology annotated pathways activated in the nodules of wild type and *sym33* mutant showed the stimulation of the functional activity of genes encoding the enzymes degrading the storage polysaccharides such as starch as well as involved in the biosynthesis of proteins, amino acids, and nucleic acids (Figure 7). Significant activation of genes encoding proteins regulating transport processes through various membranes was also shown. This reflects the difference between effective wild-type cv. SGE and ineffective SGEFix^-^-2 *(sym33)* mutant nodules. Stimulation of processes of calcium-dependent phospholipid binding suggests that signal transduction pathways activated by calcium and secondary metabolites may be involved in the regulation of effective symbiotic nodules. Stimulation of activity of pathways regulating the redox processes was also shown predominantly in effective nodules of wild type.

Analysis revealed that genes up-regulated in mutant’s nodules after cytokinin treatment are significantly enriched in processes associated with stress response (Figure 8). Among these processes, the highest rate of change was shown for ‘ribonuclease T2 activity’ which is associated with response to pathogens and abiotic stress in plants [62], and the ‘oxygen binding’ process which is important for plant stress signaling [63]. It is important to point out that genes that are up-regulated in wild nodules compared to mutant nodules are also enriched in ‘oxygen binding’.

We also found that the ‘potassium ion binding’ process was impacted by cytokinin treatment and down-regulated genes were significantly enriched in it. According to the recently published data [64], symbiosomes and vacuoles of infected cells are characterized by defects in K^+^ balance which is associated with mislocation of some plant ion channels. Aa detailed search among differentially expressed (DE) genes showed that several genes encoding potassium transporters and channels (Psat4g076320, Psat0s3462g0080) were significantly down-regulated in mutant nodules after cytokinin treatment. Similarly, the expression of Psat4g07632 and several other genes associated with ‘potassium ion binding’ processes were also decreased in wild-type nodules as compared to mutant nodules.

## 3. Discussion

It was previously shown that in *M. truncatula ipd3* and *L. japonicus cyclops* mutants the nodules remain non-infected due to the blocking of bacterial release from the infection threads and subsequent intracellular accommodation [26,29,65,66]. Similarly, pea mutant SGEFix^-^-2 (*sym33*) defective in IPD3/CYCLOPS transcription factor was impaired in the bacterial release from infection threads that resulted in non-infected nodules and disturbance in subsequent plant tissue and bacteroid differentiation [56,57,58,59]. Such mutants may be considered as a useful model for the investigation of later stages of nodule formation related to the differentiation of plant tissues and bacteroids. In addition, an altered cytokinin pattern in *sym33* mutants [55] may point towards the importance of cytokinin in the regulation of these later stages of nodulation.

To determine a set of genes involved in the regulation of later stages of nodule development connected with infection progress, intracellular accommodation, as well as plant tissue and bacteroid differentiation, the RNA-seq analysis of pea mutant SGEFix^-^-2 (*sym33*) nodules impaired in these processes compared to wild type SGE nodules was performed. To verify cytokinin’s influence on late nodule development stages, the comparative RNA-seq analysis of SGEFix^-^-2 (*sym33*) mutant plants treated with cytokinin was also conducted.

Comparative analysis of the wild type and pea *sym33* mutant nodules revealed the significant up-regulation of the genes encoding IPD3/CYCLOPS (Psat2g142200), NIN (Psat2g001120), KNOX3 (Psat6g028400), EFD (Psat7g259160), and NSP1 (Psat0s741g0280) transcription factors as well as A-type cytokinin response regulators RR11 (Psat4g192080) in wild type nodules compared with *sym33* mutant (Table S1), which is in line with our previous findings [55]. *NIN* gene (Psat2g001120) encoding transcription factor and a number of genes encoding A-type cytokinin response regulators RR11 and RR4 (Psat4g192080, Psat0s644g0040) were found among up-regulated genes in nodules of cytokinin treated *sym33* mutant (Table S2). Since the increased expression of these genes may be considered as markers of cytokinin stimulation, this indicates the expected effect of cytokinin treatment on pea mutant plants.

About 40% of the total number of up-regulated genes in wild-type nodules (compared to untreated *sym33*) matched with up-regulated genes in *sym33* mutant upon cytokinin treatment (Figure S1). Taken together with the changes in nodule number that occurred in *sym33* mutant after cytokinin stimulation, these findings suggest a significant role of cytokinin in the regulation of later stages of nodule development. Although the expression of 538 genes was altered, we can discuss only a few in this article. Among them, the genes encoding NSP1 (Psat0s741g0280), NIN (Psat2g001120), EFD (Psat7g259160), and RSD (Psat3g136520) transcription factors were shown to be up-regulated in wild type nodules compared with nodules of *sym33* mutant as well as in *sym33* mutant nodules in response to cytokinin treatment (Tables S1 and S2), thus suggesting that these transcription factors may be involved in the regulation of late stages of nodule development and induced by cytokinin. Indeed, it was recently shown that the NIN transcription factor does not only play a key role during root nodule initiation, but is also involved in the regulation of later stages of nodule development [67]. Here, we showed that stimulation of *NIN* expression as well as *NSP1, EFD,* and *RSD* at these stages may be connected with cytokinin treatment.

The *RSD* (Regulator of symbiosome differentiation, Psat3g136520) gene is involved in the regulation of the bacteroid formation. The level of *RSD* expression was higher in wild-type nodules compared to mutant nodules and up-regulated in response to cytokinin treatment of *sym33* mutant. The RSD transcription factor is required for suppression of defense responses in nodules during bacteria accommodation [68]. It controls the expression of gene encoding VAMP721a (Vesicle-associated membrane protein 721a) protein, which is important for vesicular transport. The close homolog of this protein in *Arabidopsis* is also involved in the repression of plant immunity. In the nodules of *rsd* mutant plants, the bacteroids degrade rapidly followed by brown pigment accumulation, indicating activation of the plant defense reactions [69]. Using *nin-16* weak allele mutant, it was shown that the *RSD* expression level was sufficient to suppress the development of defense reactions and early senescence in the invasion zone, but not sufficient in the fixation zone of nodules, where the level of *NIN* gene expression was significantly less. This suggests the importance of the NIN transcription factor for regulation of *RSD* expression at later stages and switching the programs controlling the development of defense reactions in different zones of nodules [67].

SEUSS-like protein (Psat6g039480) is a transcriptional adaptor in plants that provides the assembly of multimeric regulatory complexes. Gene encoding SEUSS-like protein was activated in wild-type nodules compared to *sym33* mutant nodules and in response to cytokinin treatment (Tables S1 and S2). The effect of SEUSS on the stem, leaf, and root development as well as the formation of reproductive tissues was shown in *Arabidopsis* and other plants [70]. It was suggested that this regulator may be connected with the determination of tissue specificity in plants. Within the developing flower, for example, the SEUSS protein complex physically interacts with a few MADS domain DNA-binding proteins (APETALA1, SHORT VEGETATIVE PHASE1, and AGAMOUS-LIKE24) that regulate subsequent organ development [71,72].

GATA transcription factor (Psat4g097200) was found to be significantly up-regulated in wild-type nodules compared to *sym33* mutant nodules. It is also activated in response to cytokinin treatment in *sym33* mutant nodules. In addition, another GATA transcription factor, HAN1-like (Psat6g020240), was significantly stimulated by cytokinin. Some GATA transcription factors were shown to be involved in symbiosis development in legume plants. The *MtHAN1* and *MtHAN2* genes encoding GATA transcription factors in *M. truncatula* are mainly expressed in the nodule primordia and in the infection zone in mature nodules, suggesting the participation of these transcription factors in nodulation [73]. The expression of genes encoding NCR peptides, responsible for bacteroid differentiation, is shown to be suppressed in *han1 han2* double mutant [74]. Hence, HANs may regulate nodule formation by influencing the expression and processing of small peptides including NCRs.

Indeed, NCR (Nodule cysteine-rich) secreted peptides are activated in response to rhizobial inoculation or Nod factor perception and are mainly involved in the control of bacteroid differentiation in legume plants [74,75]. An increased transcription for a set of genes encoding NCR peptides (Psat0s3945g0040, Psat2g028680, Psat2g027600, Psat2g122960, Psat0s2086g0040) was found in both sets (Tables S1 and S2). *NCR* genes may be expressed at different stages of symbiosis development. However, the expression of most of them is related to the stages when the bacteroids become mature and start fixing nitrogen, suggesting that these “late” genes are mainly involved in maintaining the functional activity of bacteroids, but not their initial development [76]. Here, it was shown for the first time that cytokinin may induce *NCR* genes related to later stages of nodule function.

Gene encoding NF-YC-like (Psat3g010480) protein was activated in wild-type nodules compared with *sym33* mutant nodules and up-regulated in response to cytokinin treatment. Components of heterotrimeric CCAAT-box-binding factor complex, the nuclear factor Y proteins (NF-Ys), composed of three subunits, NF-YA, NF-YB, and NF-YC, play an important role in plant development. NF-YA1 and NF-YA2 play an important role at early stages of rhizobial infection, but also at later stages of symbiosis, regulating nodule organogenesis in *M. truncatula* [77,78,79,80]. In addition, *M. truncatula* MtNF-YC1 and MtNF-YC2 genes are required for nodule organogenesis [81]. An important role of NF-YA1 and NF-YB1 subunits was shown in symbiotic nodule formation in *Phaseolus vulgaris* [82]. Further analysis should be performed to verify the role of pea NF-YC1 in the regulation of nodule formation.

A few genes encoding cyclins (Psat2g083400) and cyclin-like F-box proteins (Psat1g007080, Psat7g162280) showed an increased level of expression in wild-type nodules compared to *sym33* mutant nodules and activation in response to cytokinin treatment in *sym33* mutant nodules. Cyclin-like F-box proteins are one of the main components of the SCF (SKP1 (S-phase-kinase-associated protein 1), Cullin-1, F-box protein) complex that belongs to the family of E3 ubiquitin ligase. Cyclin-like F-box proteins participate in the regulation of the cell cycle associated with target protein degradation [83]. Therefore, the cyclin-like F-box proteins are mainly localized in the tissues consisting of actively dividing cells. As an example, the accumulation of transcripts of F-box proteins was found in the nodule primordia of *M. truncatula* [83]. F-box proteins may be involved in the determination of tissue specification during plant development [84]. At the same time, some F-box-like proteins can act as negative regulators of plant resistance to pathogens [85]. microRNA MiR1134 is known to suppress the F-box/RNI/FBD-like domain protein in plant defense response regulation [86].

Transcription factors of AP2/ERF (APETALA2/Ethylene responsive factor) superfamily regulate plant development and response to stress. AP2/ERF transcription factors are classified into four families, including the AP2, ERF, RAV (AP2/B3 domain), and Soloist families [87]. The gene Psat4g225040 encoding transcription factor from the AP2 family was significantly activated in wild-type nodules compared with *sym33* mutant nodules as well as in response to cytokinin treatment. The AP2/ERF transcription factors, such as ERN1 and ERN2, were shown to be essential for rhizobial infection and early stages of nodulation in legumes of legume plants [42,88]. Another AP2/ERF NNC1 (Nodule number control 1) may be a positive regulator of nodule senescence genes in mature nodules and can be silenced through miR172c as it was shown in *P. vulgaris* [89]. NNC1 directly targets and represses the early nodulin gene *ENOD40* that plays a key role in nodulation [89].

MADS-domain/AGL transcription factors belong to a large family of regulators involved in the development of plant organs. It was previously shown that these transcription factors are involved in the development of root architecture [90]. The action of MADS-box transcription factors to control the legume root and nodule architecture is suggested to be carried out through interaction with the NIN transcription regulator [91]. Gene encoding MADS-domain/AGL transcription factors (Psat4g046280) was induced in wild-type nodules compared to *sym33* mutant nodules, as well as in nodules of *sym33* mutant in response to cytokinin treatment in our experiments. Further analysis should elaborate on their role in nodule development regulation.

It is known that the cytokinin/auxin ratio is strictly controlled and plays an important role in nodule development during the legume-rhizobia symbiosis [92]. We found that the cytokinin treatment may stimulate the expression of SHI/STY (Short internode/stylish) (Psat0s133g0120) gene, the closest homolog of STY1 from *L. japonicus*. SHI/STY transcription factors participate in the development of the generative organs and are important for the correct specification of tissue identity and differentiation in plants [93,94,95]. As SHI/STY may fulfill a similar function in various plants and mosses, we can talk about a conservative role of SHI/STY in tissue. Their participation in the development of nodules in *Medicago* was shown to be connected to the regulation of YUCCA enzymes, involved in the local biosynthesis of auxin. *YUCCA1* (Psat6g030600) gene was also found among differentially expressed genes in nodules of *sym33* mutant treated with cytokinin. Moreover, up-regulation in wild type and cytokinin treated mutant was shown for a set of genes encoding auxin-regulated proteins. Since the SHI/STY may be the direct regulators of auxin biosynthesis, they are important for the early stages of nodule organogenesis but seem to be also required for subsequent nodule development [96]. In *L. japonicus*, the SHI/STY was found to promote the differentiation of nodule structure, including an increase in cell size and the formation of vascular bundles [97].

In our experiments, the treatment of wild-type plants with cytokinin did not result in visible morphological changes of nodule structural features as the light microscopy and TEM showed. However, treatment of *sym33* mutant plants with cytokinin according to the results of TEM analysis may induce partial degradation of bacteria in the infection threads. Moreover, it may stimulate the activation of callose and starch deposition in the cells of mutant as analysis of gene ontology pathways showed. Although cytokinin stimulated the additional formation of nodules in *sym33* mutant, it seems that the development of bacteria inside infection threads in these nodules was disturbed. Comparative transcriptomic analysis of the wild type and mutant mature nodules showed stimulation of defense reactions, but this stimulation was stronger in nodules of cytokinin treated *sym33* mutant compared with non-treated. As an example, the gene (Psat7g031360) encoding RING-H2 zinc finger protein was up-regulated in wild-type nodules compared with *sym33* mutant nodules, but its expression level was significantly increased in response to cytokinin treatment of mutant plants. RING-H2 zinc finger protein and E3 ubiquitin-protein ligase are shown to be positive regulators of plant resistance to stress [98]. All the above-mentioned suggest that cytokinin may be an important regulator of plant resistance to infection. This balance is tightly regulated and may be disturbed by cytokinin regulating defense reactions in plants.

Therefore, transcriptomic profiling of wild-type and mutant nodules, as well as nodules in plants treated with cytokinin, resulted in the identification of a number of differentially expressed genes. This demonstrates the importance of the IPD3/CYCLOPS transcription factor for the regulation of later stages related to nodule differentiation and its interplay with cytokinin at these stages. We found many genes that may be involved in the regulation of the intracellular accommodation of bacteria and subsequent plant tissue and bacteroid differentiation among them. Further analysis should be performed to verify the role of these regulators in the control of late stages of nodule morphogenesis in pea.

## 4. Materials and Methods

### 4.1. Bacterial Strains and Inoculation

The *Rhizobium leguminosarum biovar viciae* CIAM 3841 strain was cultured at 28 °C on TY agar medium [99] supplemented with 0.6 mg/mL streptomycin. For the preparation of inoculum, bacteria were incubated in liquid B^-^ medium [100]. The optical density of the suspension at 600 nm (OD_600_) was adjusted to 0.5–0.7.

### 4.2. Plant Material and Growth Conditions

*Pisum sativum L.* SGE cultivar and SGEFix^-^-2 (*sym33-3*) mutant seeds from a collection of ARRIAM (St. Petersburg, Russia) were sterilized with sulfuric acid for 10 min, washed with sterile water 4 times, transferred to plates with 0.8% water agar, and germinated at room temperature in the dark for 4–5 days. After germination, the seedlings were transferred to the pots with vermiculite, saturated with Jensen’s medium, and grown in a climatic chamber (Binder, Germany) at 21 °C with cycles of 16 h light/8 h dark and humidity 64%. Pea seedlings were inoculated with 2 mL of *R. leguminosarum* bv*. viciae* CIAM 3841 per plant on the next day after transfer. One day after, half of the plants were treated with 6-BAP, at a final concentration of 10 μm and a volume of 50 mL per pot (diluted with sterile distilled water). Treatment was carried out every other day, and control plants were watered in the same volume with sterile distilled water. For gene expression analysis, the nodules were harvested after 14 days.

### 4.3. Material Fixation

The nodules were fixed in a freshly prepared solution of 2% paraformaldehyde (Sigma-Aldrich, MO, USA) in 1/3 of the concentration of MTSB buffer (50 mM PIPES (pH 6.9) (Amresco, OH, USA); 5 mM MgSO_4_ × 7H_2_O; 5 mM ethylene glycol-bis (β-aminoethyl ether)—N, N, N‘, N’-tetraacetic acid (Sigma, MO, USA) with the addition of 0.25% glutaraldehyde (solution Grade I, 25% in H_2_O. Sigma-Aldrich, MO, USA), 0,3% Twin-20 (Amresco, OH, USA), 0,3% Triton-X-100 (Amresco, OH, USA). For optimal penetration of the fixative, the air from the tissue was evacuated three times for 7 min at 0.9 bar using a ME 1 vacuum pump (Vacuubrand, Germany) and left overnight at +4 °C. Then, the material was washed with PBS buffer (0.137 M NaCl, 0.0027 KCl, 0.01 M Na_2_HPO_4_, 0.0018 M KH_2_PO_4_, pH 7.4).

### 4.4. Light and Transmission Electron Microscopy

The nodules were fixed in solution of 2.5% (*v*/*v*) glutaraldehyde (EMS, Hatfield, PA, USA) and 2% (*w*/*v*) formaldehyde (EMS, Hatfield, PA, USA) in 0.1 M cacodylate buffer (pH 7.4) overnight and postfixed in ice-cold 2% OsO4 (EMS, Hatfield, PA, USA) in the same buffer for 1.5 h. Samples were dehydrated in ethanol series and acetone and embedded in EmBed 812 epoxy resin (EMS, Hatfield, PA, USA). Semithin (1 μm) and ultrathin sections were made using ultratome EM UC7 (Leica, Vienna, Austria). Semithin sections were stained with 20% solution of Epoxy tissue stain with toluidine blue and basic fuchsin (EMS, Hatfield, PA,USA) in 50% ethanol and observed under light microscope DM2500 (Leica, Vienna, Austria). Ultrathin sections were stained on grids with 2% uranyl acetate water solution and Reynold’s lead citrate. Images were taken using the transmission electron microscope JEM-1400 (Jeol, Tokyo, Japan) equipped with a side camera Veleta (Olympus, Tokyo, Japan) at 80 kV.

### 4.5. Isolation of RNA and Preparation of Libraries

According to the manufacturer’s protocol, the total RNA was isolated from the nodules of inoculated or treated with cytokinin plants using the PureZol reagent (Bio-Rad Laboratories, Hercules, CA, USA). To remove genomic DNA, DNAseI treatment (Thermo Fisher Scientific, Waltham, MA, USA) was used.

To prepare libraries for the experiment with SGE Fix^-^-2 (*sym33-3*), we used NEBNext^®^ Poly (A) mRNA Magnetic Isolation Module (E7490) purification kit, NEBNext^®^ Ultra ™ II Directional RNA Library Prep Kit for Illumina^®^ (E7760), and the NEBNext^®^ Multiplex Oligos primer set for Illumina^®^ (Index Primers Set 2) (E7500S) (New England Biolabs, Rowley, MA, USA). At all stages, the material was cleaned using AMPure XP beads (Beckman Coulter, Brea, CA, USA).

### 4.6. Illumina Sequencing and Data Analysis

Three groups of libraries representing three independent biological experiments (4–5 plants for one replication of one treatment option) were sequenced on a NovaSeq 6000 (Illumina, San Diego, CA, USA) with single-end 100 bp reads according to the manufacturer’s protocol. The resulting reads were trimmed off Illumina adapters. Additionally, read ends were trimmed by sequencing quality (phred33 score < 20) using Trimmomatic [101]. To obtain read counts per both transcript and gene, fastq files were further processed with RSEM package [102] using transcriptome generated from Pisum sativum genome v1a [103]) as a reference. The differential expression was performed on the resulting raw count matrix of 9 individual samples using the DESeq2 R package [104]. Transcripts were considered to be differentially expressed with the adjusted *p*-value < 0.05 and absolute value of log2 (fold change) > 2. Gene ontology [105] gene set enrichment analysis was executed using the GSEABase R package [106] with cutoff values: odds ratio > 2, and *p*-value < 0.05.

### 4.7. Statistical Analysis

Statistical analysis of root length and number of nodules was based on the results of three independent experiments (4–5 plants per one variant were used). The error bars represent the mean ± SEM of three repeats. The asterisks indicate statistically significant differences based on Student’s *t*-test (* *p* < 0.05; ** *p* < 0.01).

## Data Availability

Not applicable.

## Supplementary Materials

The following materials are available online at https://www.mdpi.com/article/10.3390/plants11010056/s1, Table S1: Analysis of differential gene expression in pea nodules of cv. SGE wild type (wt) and SGEFix^-^-2 (*sym33*) mutant; Table S2: Analysis of differential gene expression in pea nodules of SGEFix^-^-2 (*sym33*) mutant plants untreated or treated with cytokinin; Figure S1: The graphical output of differential gene expression in pea nodules of cv. SGE wild type and SGEFix^-^-2 (*sym33*) mutant as well as in pea nodules of SGEFix^-^-2 (*sym33*) mutant plants untreated or treated with cytokinin based on Venna diagram using cutoff threshold equal log2 fold change value > 2 and *p* adjusted value < 0.05.
