# Supplementary figures and images for: Regulation of the Later Stages of Nodulation Stimulated by IPD3/CYCLOPS Transcription Factor and Cytokinin in Pea Pisum sativum L
Source: Plants (Basel). 2021 Dec 25;11(1):56. doi: 10.3390/plants11010056 (PMC8747635; doi:10.3390/plants11010056)

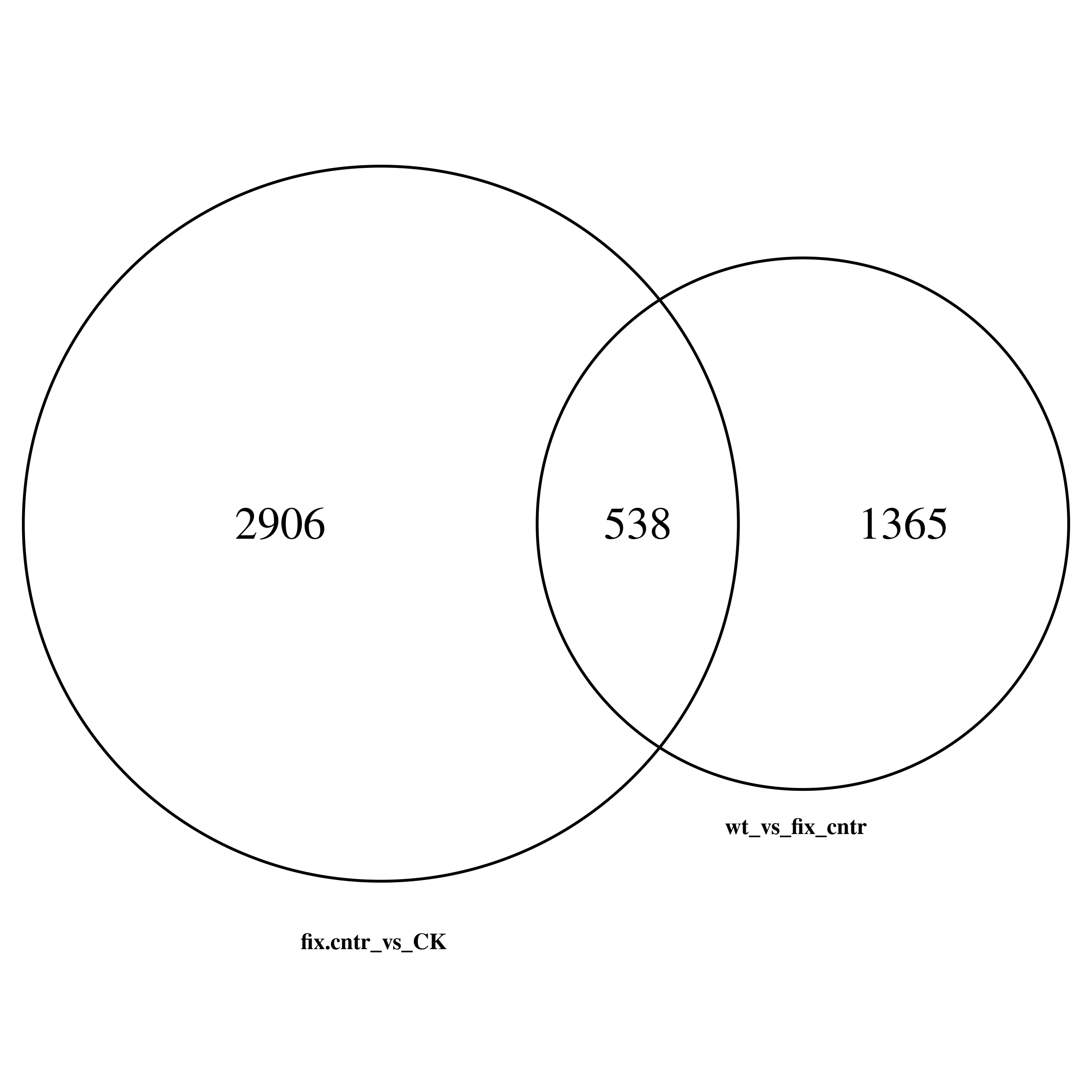

Supplement: Supplementary file 1 [file plants-11-00056-s001.zip › Figure 1S.png]
